# Exploring Cariprazine’s Potential in Late-Stage Schizophrenia Treatment

**DOI:** 10.1192/j.eurpsy.2024.453

**Published:** 2024-08-27

**Authors:** P. Falkai, R. Csehi, K. Acsai, G. Németh

**Affiliations:** ^1^Department of Psychiatry and Psychotherapy, University Hospital of Munich Ludwig Maximilian, Munich, Germany; ^2^Global Medical Division, Gedeon Richter Plc, Budapest, Hungary

## Abstract

**Introduction:**

Schizophrenia is a chronic neuropsychiatric disorder that often requires long-term pharmacotherapy to manage symptoms and prevent relapse. There are important clinical differences between early-stage versus late-stage schizophrenia, like the predominant symptomatology. In later stages, negative, cognitive, and anxiety/depressive symptoms dominate the clinical picture, with relapses further potentiating the emergence of positive symptoms. Therefore, it is crucial to establish the efficacy of an antipsychotic medication in the later stages of schizophrenia as well. Cariprazine is a novel dopamine D3-preferring D3/D2 receptor partial agonist that has shown efficacy in treating schizophrenia across the symptom spectrum.

**Objectives:**

The aim of this poster is to present the findings of cariprazine’s efficacy in treating late-stage schizophrenia, especially in symptoms that are more commonly occurring in this phase of the disorder.

**Methods:**

This poster reports the results of a post-hoc pooled analysis of three 6-week, double-blind, placebo-controlled trials (NCT01104766, NCT01104779, NCT00694707) that assessed the efficacy of cariprazine in schizophrenia. The primary outcome was the change in Positive and Negative Syndrome Scale (PANSS) Total Scores from baseline to endpoint. The analysis focused on patients with late-stage schizophrenia (defined as having an illness-duration of more than 15 years) who received cariprazine at doses between 1.5 mg/day to 6.0 mg/day. The changes in PANSS-derived Marder Factor Scores for Negative, Disorganised Thought (i.e., Cognitive) and Anxiety/Depression symptoms were further examined. The least square mean differences (LSMDs) between cariprazine and placebo groups were calculated using mixed-models for repeated measures (MMRM).

**Results:**

Altogether, 128 placebo-, and 286 cariprazine-treated patients were identified as having schizophrenia for more than 15 years. The mean age of patients was about 45 years, while the mean illness-duration was about 24 years. The mean baseline PANSS scores were the same between the two groups. In the late-stage schizophrenia population, at Week 6, cariprazine yielded statistically significantly greater reductions on the PANSS Total Score (LSMD -6.7, p<0.01). Cariprazine further showed superiority over placebo in reducing negative (LSMD -1.4, p<0.05), disorganised thought (LSMD -1.3, p<0.01), and anxiety/depression (LSMD -0.9, p<0.05) symptoms.

**Conclusions:**

Cariprazine showed efficacy in treating patients with late-stage schizophrenia. It improved overall schizophrenia symptoms, as well as the negative, cognitive and anxiety/depression symptoms that are more prevalent in this phase of the disorder.

**Disclosure of Interest:**

P. Falkai Consultant of: Janssen-Cilag, AstraZeneca, Lilly, and Lundbeck, Speakers bureau of: AstraZeneca, Bristol Myers Squibb, Lilly, Essex, GE Healthcare, GlaxoSmithKline, Gedeon Richter, Janssen Cilag, Lundbeck, Otsuka, Pfizer, Servier, and Takeda, R. Csehi Employee of: Gedeon Richter Plc, K. Acsai Employee of: Gedeon Richter Plc, G. Németh Employee of: Gedeon Richter Plc

